# Impact of possible errors in natural language processing-derived data on downstream epidemiologic analysis

**DOI:** 10.1093/jamiaopen/ooad111

**Published:** 2023-12-27

**Authors:** Zhou Lan, Alexander Turchin

**Affiliations:** Center for Clinical Investigation, Brigham & Women’s Hospital, Boston, MA 02115, United States; Harvard Medical School, Boston, MA 02115, United States; Harvard Medical School, Boston, MA 02115, United States; Division of Endocrinology, Brigham & Women’s Hospital, Boston, MA 02115, United States

**Keywords:** natural language processing, epidemiology, outcomes research, Monte Carlo method, electronic health record

## Abstract

**Objective:**

To assess the impact of potential errors in natural language processing (NLP) on the results of epidemiologic studies.

**Materials and Methods:**

We utilized data from three outcomes research studies where the primary predictor variable was generated using NLP. For each of these studies, Monte Carlo simulations were applied to generate datasets simulating potential errors in NLP-derived variables. We subsequently fit the original regression models to these partially simulated datasets and compared the distribution of coefficient estimates to the original study results.

**Results:**

Among the four models evaluated, the mean change in the point estimate of the relationship between the predictor variable and the outcome ranged from −21.9% to 4.12%. In three of the four models, significance of this relationship was not eliminated in a single of the 500 simulations, and in one model it was eliminated in 12% of simulations. Mean changes in the estimates for confounder variables ranged from 0.27% to 2.27% and significance of the relationship was eliminated between 0% and 9.25% of the time. No variables underwent a shift in the direction of its interpretation.

**Discussion:**

Impact of simulated NLP errors on the results of epidemiologic studies was modest, with only small changes in effect estimates and no changes in the interpretation of the findings (direction and significance of association with the outcome) for either the NLP-generated variables or other variables in the models.

**Conclusion:**

NLP errors are unlikely to affect the results of studies that use NLP as the source of data.

## Introduction

Widespread adoption of electronic health records (EHRs) has led to an exponential growth of healthcare data.^1^ With this growth come opportunities to leverage these immense amounts of information to streamline processes, decrease costs, discover new treatments, and improve patient outcomes. One distinct component of EHR data is narrative documents. This important data category, which can include clinicians’ notes, radiology reports, and operative summaries, offers richness of detail, nuance of medical uncertainty, and the logic of clinicians’ decision-making.

Modern artificial intelligence (AI) technologies for analysis of text, collectively known as natural language processing (NLP), provide a key to unlock this treasure trove of information.^2–4^ Information supplied by NLP tools has been used in a broad range of medical data analyses, including predictive modeling, analyses of social determinants of health, and health outcomes research,^5–7^ and has often provided unique perspectives not available from other data sources^8^.

An important aspect of NLP-based information extraction is that, unlike for structured data, the process of translating the raw data into discreet analytical fields is not usually perfectly accurate. Recall and precision of state-of-the-art NLP systems vary widely depending on the application, often falling in the range between 60% and 80%.^8–13^ The effect of these errors on the results of clinical investigations that utilize NLP-generated data is not known. We therefore conducted a study of possible effects of NLP errors on the results of multivariable regression models in epidemiologic clinical research.

## Methods

### Study design

We conducted a Monte Carlo simulation analysis based on data from three previous epidemiologic studies to investigate the impact of possible errors in NLP-derived data on the results of multivariable regression models.

### Study settings

All studies included in the analysis were conducted at Mass General Brigham (MGB), a large integrated healthcare delivery network in Massachusetts, which includes several community and specialty hospitals and affiliated outpatient primary care and specialty practices. MGB has maintained a comprehensive EHR system that incorporates a wide range of structured and narrative data sources since 2000. The source of data for all studies used in this analysis was the MGB EHR.

### Contributing studies

The present study used data from three previous studies: Turchin et al.^14^, Chang et al.^15^, and Brown et al.^16^ (Table 1). Turchin et al.^14^ investigated the association between non-acceptance (decline) of insulin therapy recommendation by patients and time to glycemic control (defined as HbA1c <7.0%). The primary predictor variable in this study, non-acceptance of insulin therapy recommendation, was identified using NLP analysis of EHR provider notes. The multivariable analysis from Turchin et al.^14^ included in the present study used a propensity-weighted Cox proportional hazards model, which adjusted for socio-demographic characteristics, health status, and treatment. Chang et al.^15^ explored the relationship between patient-provider discussions of bariatric surgery and subsequent weight changes and receipt of bariatric surgery. The primary predictor variable in this study, documentation of bariatric surgery discussions in EHR provider notes, was identified using NLP analysis. Two multivariable analyses from Chang et al.^15^ were included in the present study: a hierarchical mixed linear regression model with random intercepts, which investigated the association between bariatric surgery discussion and changes in BMI (primary analysis in the original study); and a logistic regression model, which analyzed the relationship between bariatric surgery discussion and receipt of bariatric surgery (a secondary analysis in the original study). Both models adjusted for socio-demographic characteristics and relevant comorbidities. Brown et al.^16^ explored the relationship between non-acceptance of statin therapy recommendation by patients and time to achieving low-density lipoprotein cholesterol (LDL) levels <100 mg/dL. The multivariable analysis from Brown et al.^16^ included in the present study used a Cox proportional hazards model, which adjusted for socio-demographic characteristics and relevant comorbidities. All NLP tools used to generate data for the original studies were developed using the Canary platform,^17^ publicly available at http://canary.bwh.harvard.edu/. Recall of the NLP tools in these studies ranged from 0.88 to 1.0 and precision from 0.76 to 0.95 (Table 1).

**Table 1.** Studies contributing data to the analysis.

Reference	NPL-derived medical variable	Outcome	Statistical model	Positive predictive value (PPV)	Negative predictive value (NPV)	Sensitivity
Turchin et al.^14^	Non-acceptance of insulin therapy by patients	Time to HbA1c <7.0%	Propensity score weighted Cox model	0.95	0.99	1.00
Chang et al.^15^	Patient-provider discussions of bariatric surgery	Change in BMI	Linear mixed model	0.76	0.99	0.89
		Receipt of bariatric surgery	Logistic regression			
Brown et al.^16^	Non-acceptance of statin therapy by patients	Time to LDL <100 mg/dL	Cox model	0.78	0.99	0.88

The table provides the reference of the contributing studies and their key information inclusive of NPL-derived medical variable, outcome, statistical model, PPV, NPV, and sensitivity.

### Semi-synthetic data-based evaluation

**Figure 1.**
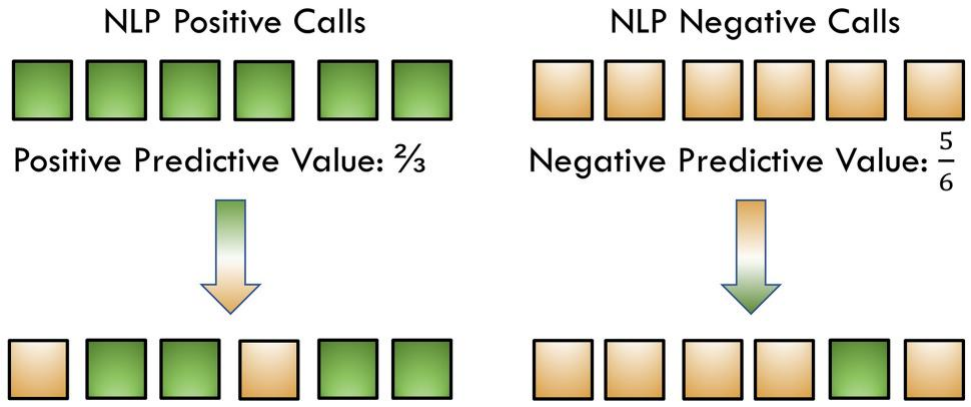
Illustration of generation of semi-synthetic data.

To provide a concise illustration, we used the term “original data” to refer to the data used in one of the three contributing studies. Similarly, the terms “NLP-derived variable” and “outcome” referred to their corresponding terms in one of the three studies. Accuracy of the NLP-derived variable is typically not perfectly accurate. Positive calls may contain false positives (true negatives), and negative calls may contain false negatives (true positives). In real settings, the true underlying values (ie, not NLP-derived) cannot be accessed. To assess the impact of NLP-derived data on downstream analyses, we simulated the underlying true values using the following approach. Since the positive predictive value (PPV) and negative predictive value (NPV) are defined as PPV = (number of true positives)/(number of positive calls) and NPV = (number of true negatives)/(number of negative calls), respectively, we performed the following two manipulations for each simulation: a) Among the NLP-derived positive calls, we randomly selected N_P_ × (1 − PPV) calls and changed them to negative calls, where N_P_ is the number of positive calls; b) Among NLP-derived negative calls, we randomly selected N_N_ × (1 − NPV) calls and changed them to positive calls, where N_N_ is the number of negative calls (Figure 1). Following the spirit of Monte Carlo simulation, we performed 500 simulations to mimic all possible scenarios. Since all other variables were kept constant, we referred to the data after manipulation as “semi-synthetic data.” The values of PPV and NPV used for data generation are provided in Table 1. For each of the three studies, we combined the synthetic NLP-derived predictor variable data with the original confounder data (ie, socio-demographic characteristics and relevant comorbidities) and fit the resulting dataset to the corresponding statistical model. For Cox regression, we used the function *coxph()* in the R package “survival”^18^ to generate hazard ratios for each variable. For the logistic regression, we used the function *glm*() in the R package “stats” to generate odds ratios for each variable. For the linear mixed model, we used the function *lmer*() in the R package “lme4”^19^ to generate fixed effect estimates for each variable. Using the R package “*performance*,”^20^ we have checked the model assumption of each run of the simulation study. The simulation runs that did not follow the model assumptions have been excluded from the analysis.

We used boxplots to visualize the above estimates and compare them to the ones obtained from the original data. The point estimation comparison was used for this measurement. For each variable and model, we also measured the probability (among the 500 simulations) of the estimate changing its direction (sign) and arising or disappearing statistical significance (*P*-value < .05) of the relationship between the variable and the model outcome.

## Results

### Impact on the relationship between the NLP-generated variable and study outcome

The impact of possible NLP errors on the relationship between the NLP-derived variable and study outcome is summarized in Table 2. In Table 2, the mean % change of the point estimate provided the percentage change between the original estimate and the point estimate for the 500 simulations. Among the four analyses of the relationships the percentage change between the original estimate and the point estimate over all simulations and covariates for the 500 simulations ranged from −21.9% to 4.12% (Table 2). Given that the NLP-derived variables had significant effects on the outcomes, we reported the probability that the significance was eliminated among the simulations (Table 2). In one of the four analyses 12% of the 500 simulations showed the relationship between the NLP-derived variable and the study outcome lose its statistical significance; in the other three analyses the relationship maintained its statistical significance in all 500 simulations. None of the 500 simulations showed the relationship between the NLP-derived variable and the corresponding study outcome change direction (sign of the regression coefficient).

**Table 2.** Impact of possible NLP errors on the relationship between the NLP-derived variable and study outcome.

Original study	Outcome	Original point estimate	Mean % change of the point estimate (IRQ)	Original *P*-value	Significance eliminated, mean % simulations
Turchin et al.^14^	Time to HbA1c <7.0%	HR: 0.90	0.61 (0.46, 1.68)	.0122	12
Chang et al.^15^	Change in BMI	Fixed effect: −1.43	−4.03 (−7.22, −0.714)	<.001	0
	Receipt of bariatric surgery	OR: 10.23	−21.90 (−24.3, −19.8)	<.001	0
Brown et al.^16^	Time to LDL <100 mg/dL	HR: 0.57	4.12 (3.36, 4.99)	<.001	0

### Impact on the relationship between non-NLP-generated variables and study outcome

Similar summarizations to investigate the impact of possible NLP errors on the relationships between other confounder variables and study outcome are given in Table 3, and the results are summarized over all confounders and replications. Among the 65 confounders in the four statistical models included in the study, none changed the direction of their relationship with the corresponding study outcome in any of the 500 simulations. The median change of the point estimate from the original study ranged from 0.039% to 2.27% (Table 3). In one of the four analyses none of the confounders that did not previously have a statistically significant relationship with the outcome acquired statistical significance in any of the 500 simulations. In the remaining three analyses, between 0.63% and 1.51% of the simulations of the relationships between confounder variables and study outcomes that did not previously have statistical significance, acquired it. In two of the analyses none of the confounder variables that had a statistically significant relationship with the study outcome lost statistical significance in any of the 500 simulations. In the remaining two analyses, between 0.96% and 9.25% of simulations showed a loss of statistical significance.

**Table 3.** Impact of possible NLP errors on the relationships between other confounder variables and study outcome.

Original study	Outcome	Confounders	Change direction	Mean % change of the point estimate (IQR)	Significance acquired, mean % simulations	Significance eliminated, mean % simulations
Turchin et al.^14^	Time to HbA1c <7.0%	14	0	0.039% (−0.004%, 0.01%)	0	0
Chang et al.^15^	Change in BMI	14	0	2.27% (−2.331%, 4.237%)	1.37	0
	Receipt of bariatric surgery	14	0	0.72% (−0.11%, 2.05%)	1.51	9.25
Brown et al.^16^	Time to LDL <100 mg/dL	23	0	0.27% (−0.024%, 0.603%)	0.63	0.96

**Figure 2.**
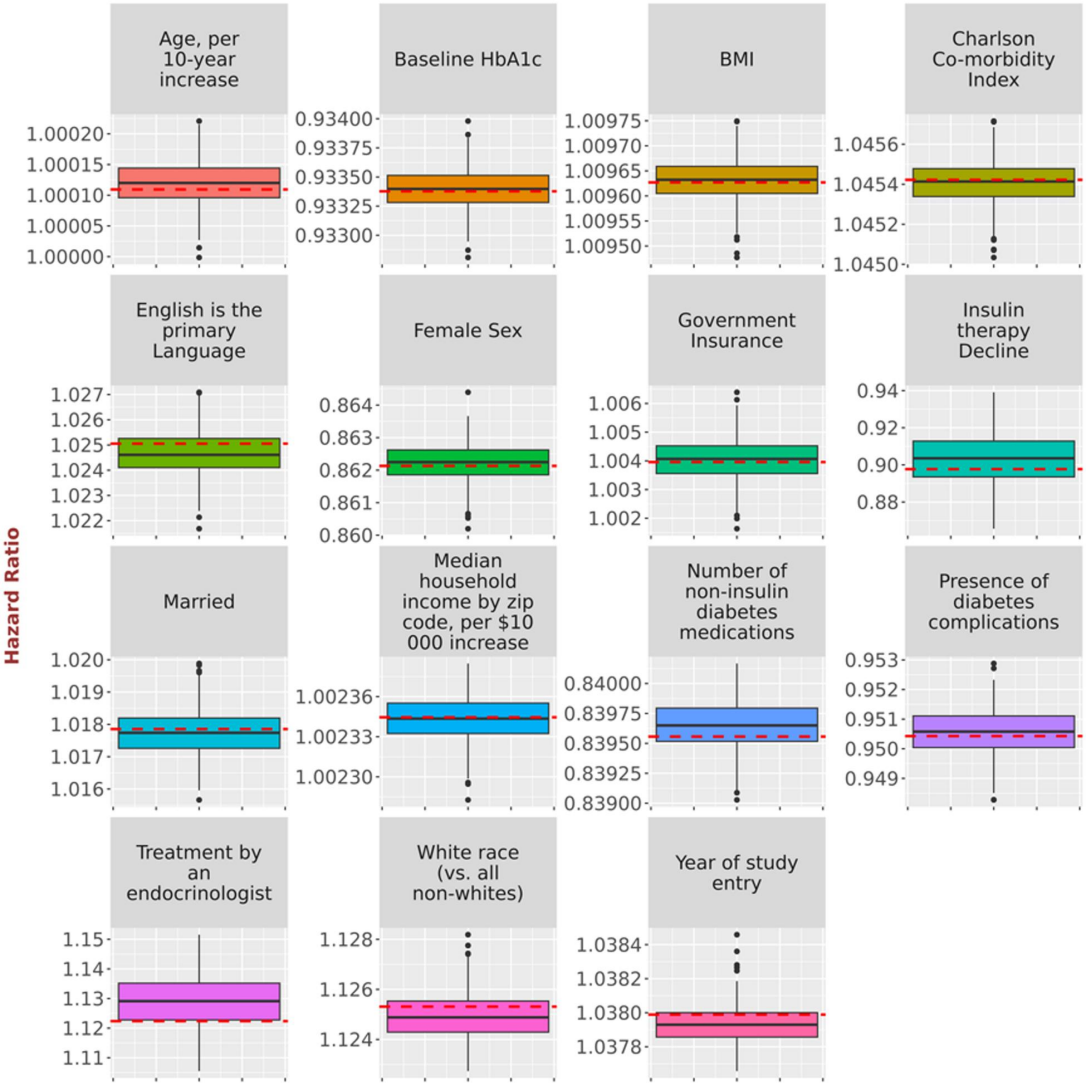
Comparison of point estimates of hazard ratios for the relationship with time to HbA1c < 7.0% in the simulations of possible NLP errors vs the original study in Turchin et al.^14^ Dashed horizontal lines represent the original point estimate. Solid horizontal lines represent in the boxplot the median point estimate for the 500 simulations. Shaded area represents the interquartile range for the 500 simulations. Vertical line and the shaded circles represent the entire range of the estimates for the 500 simulations.

While Tables 2 and 3 provide overall summarization of the results, we present a comparison of point estimates derived from the original data and synthetic data simulations using boxplots (Figures 2, 3A and 3B, and 4), allowing us to inspect the impact of NLP errors on the point estimation for each coefficient. Figure 2 provides the comparison of point estimates of hazard ratios for the relationship with time to HbA1c <7.0% in the simulations of possible NLP errors vs the original study in Turchin et al.^14^Figure 3A provides the comparison of point estimates of regression coefficients for the relationship with BMI change in the simulations of possible NLP errors vs the original study in Chang et al.^15^Figure 4 provides the comparison of point estimates of hazard ratios for the relationship with time to LDL <100 mg/dL in the simulations of possible NLP errors vs the original study in Brown et al.^16^ The variability of the point estimates obtained from the 500 synthetic data simulations is represented by the range of the boxplots. Discrepancies can be observed by comparing these results to the red line, which represents the estimates from the original data.

**Figure 3.**
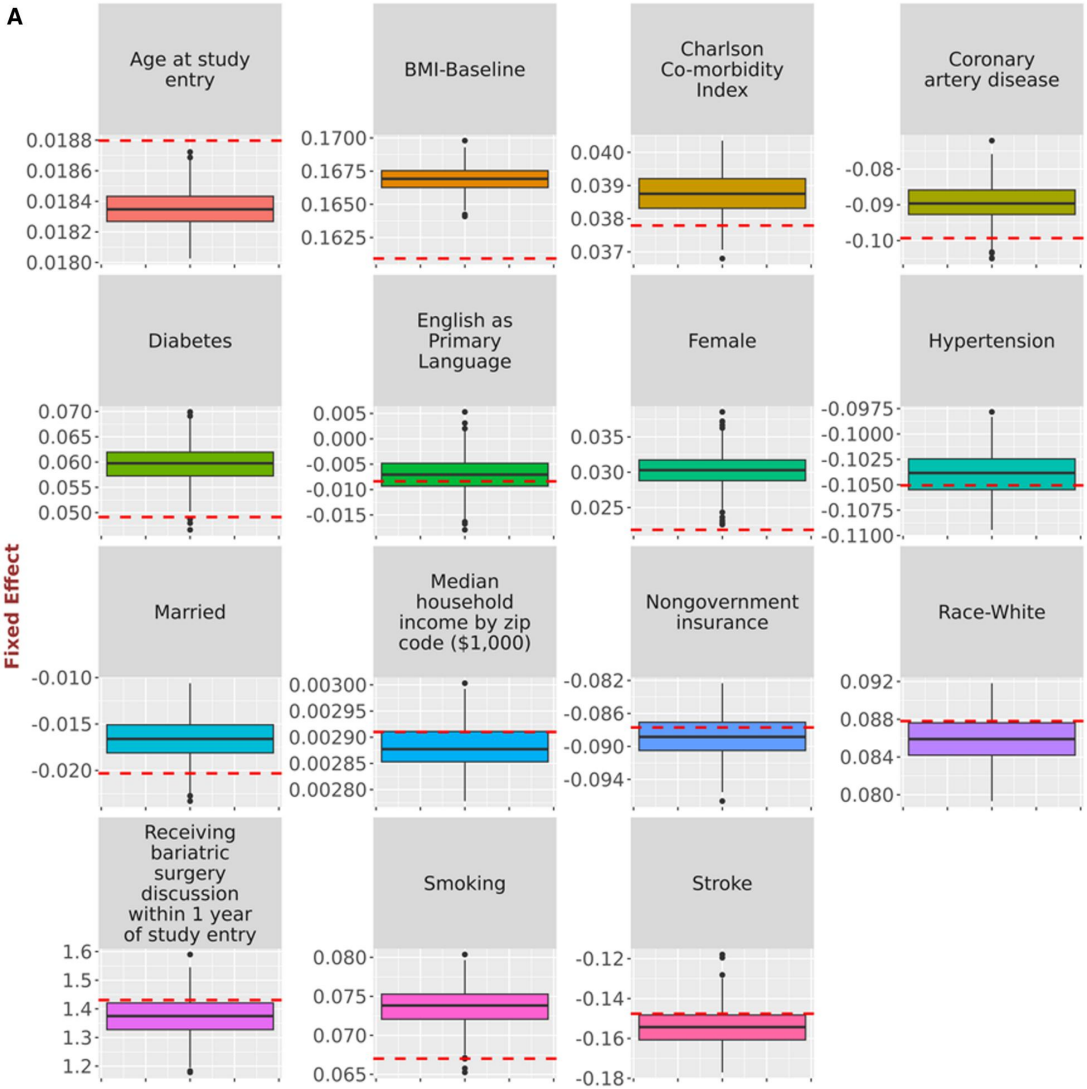
Comparison of point estimates of (A) regression coefficients for the relationship with BMI change and (B) odds ratios for the relationship with receipt of bariatric surgery in the simulations of possible NLP errors vs the original study in Chang et al.^15^ Dashed horizontal lines represent the original point estimate. Solid horizontal lines represent in the boxplot the median point estimate for the 500 simulations. Shaded area represents the interquartile range for the 500 simulations. Vertical line and the shaded circles represent the entire range of the estimates for the 500 simulations.

**Figure 3.**
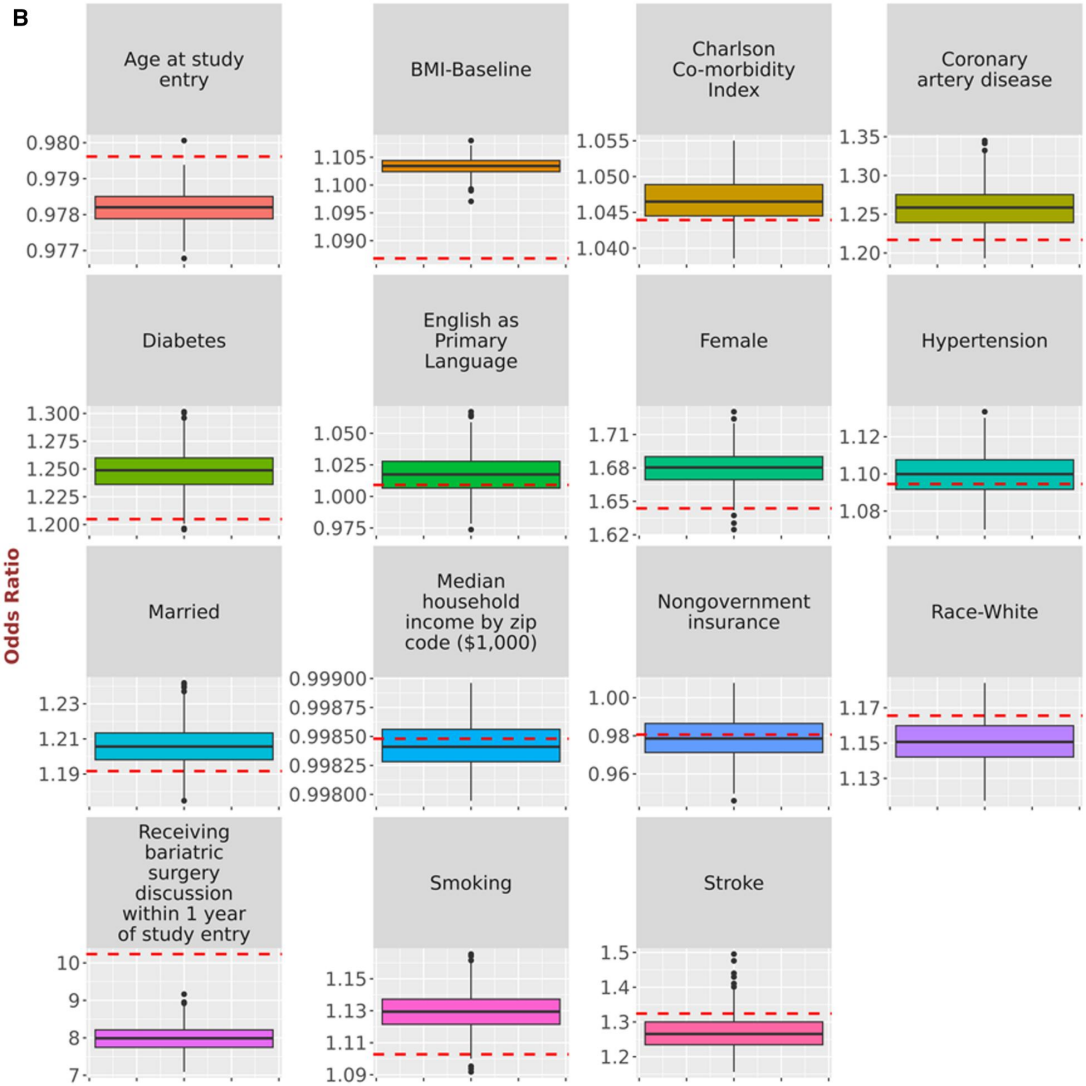
(Continued)

**Figure 4.**
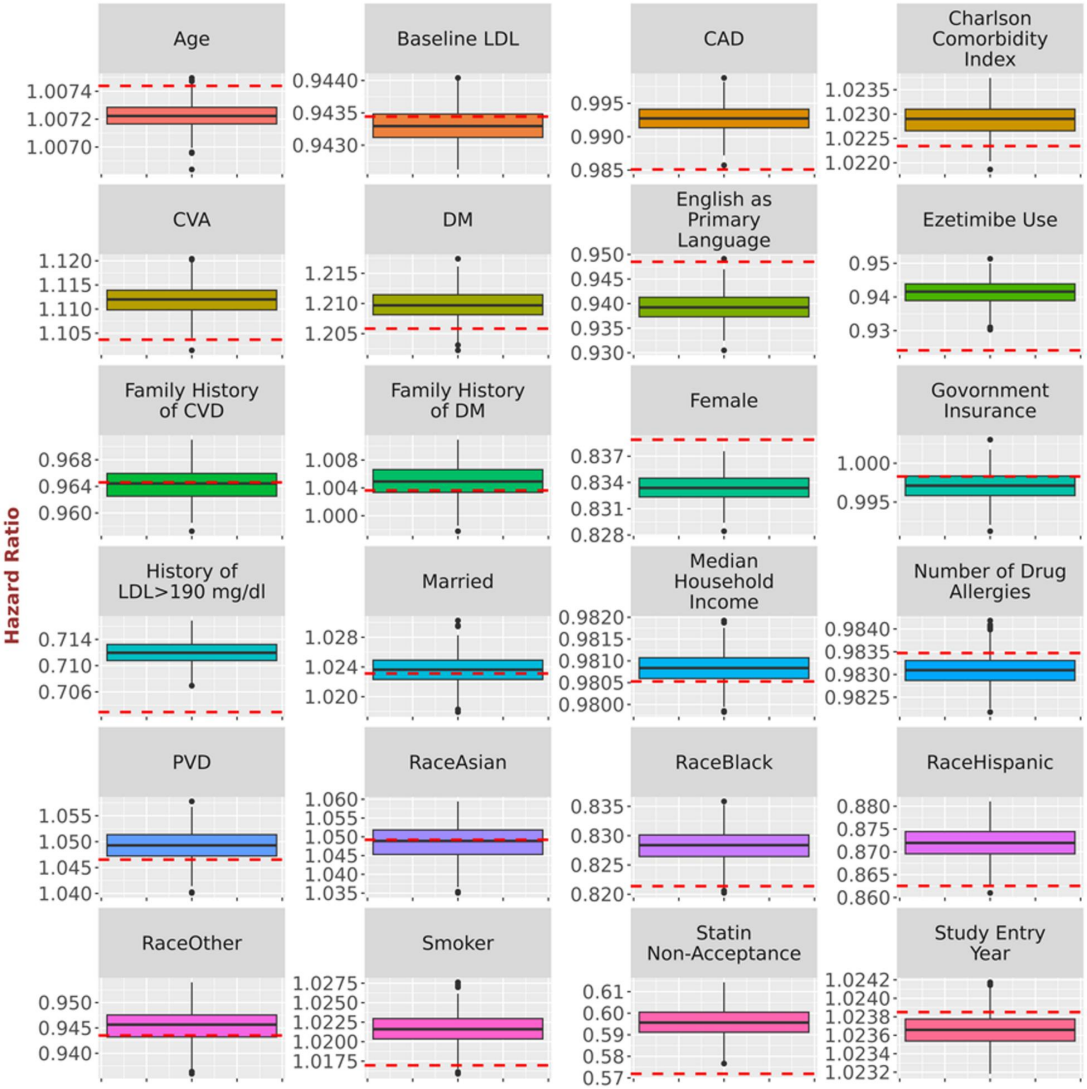
Comparison of point estimates of hazard ratios for the relationship with time to LDL <100 mg/dL in the simulations of possible NLP errors vs the original study in Brown et al.^16^ Dashed horizontal lines represent the original point estimate. Solid horizontal lines represent in the boxplot the median point estimate for the 500 simulations. Shaded area represents the interquartile range for the 500 simulations. Vertical line and the shaded circles represent the entire range of the estimates for the 500 simulations.

## Discussion

In this simulation study of several NLP-based multivariable analyses, the impact of possible NLP errors on the regression model results was modest. Associations of all of the NLP-derived variables with the corresponding study outcomes retained statistical significance in all or overwhelming majority of the simulations, and median difference in point estimates did not exceed 25%.

This finding has significant implications for epidemiological studies and outcomes research that use NLP-derived data. Human language is incredibly rich, and it is simply not feasible to program—whether by hand or through machine learning methods—all possible permutations that can carry a particular meaning. Furthermore, language is often ambiguous and therefore perfectly accurate interpretation may not even theoretically be feasible. If these inaccuracies translate into dramatically different results in downstream analyses that utilize NLP-derived data, then reliability of the corresponding study results and their implications for patient care would be called into question. The results of this study reassuringly demonstrate that the impact of NLP errors on the results of regression models widely used in epidemiological studies is limited. These results were independent of the regression model used and included some of the most commonly used models—logistic regression, linear regression, mixed models, and Cox proportional hazards models. They will therefore generalize on a large number of observational studies that utilize NLP-derived data.

The role of NLP in epidemiologic and observational studies continues to grow. It has been used in studies across a broad swathe of clinical fields, ranging from radiology to surgery to multiple subspecialties of internal medicine.^8^^,^^21–23^ As the amount of available data continues to explode and new and better technologies are developed, its importance is set to continue to increase. The finding of the present study that errors in NLP-based data acquisition have only a very limited impact on downstream regression analyses removes another important barrier to acceptance of NLP as an essential data source for clinical research.

It is important to note that the present analysis was limited to regression models, where statistical significance (or lack thereof) of the relationship between predictor/confounder variables and the outcome is the most critical finding. Other applications of NLP-derived data where the precise value of the point estimate is essential, such as predictive modeling, may be more strongly affected by NLP errors. For these potentially more error-sensitive applications it would be important to conduct sensitivity analyses similar to this study that would evaluate the potential impact of NLP errors and allow the readers to judge the reliability and calibration of the model.

Monte Carlo simulation methods are widely used in the community of statistics and machine learning to simulate situations that are impractical or impossible to replicate in real-world settings. In medical EHR research, for example, the underlying medical condition may not be readily discernible from the available medical reports. While Monte Carlo simulation has been employed in some medical scenarios,^24^^,^^25^ few studies have investigated the impact of possible errors in NLP-derived data on downstream epidemiologic analysis using this approach. Our paper, together with Brown et al.,^16^ may be among the first papers to systematically examine this issue.

State-of-the-art NLP systems typically achieve recall and precision rates in the range between 60% and 80%.^8–13^ However, our evaluation results suggested that the impact of possible false positives on the model estimates of all variables in the model was minor. For most of the non-NLP-derived variables, their robustness may be attributed to their strong marginal effects. For most of the NLP-derived variables, their robustness may be explained by the existing correlations, which are unaffected by changes in possible false positives.

The present study had a number of strengths. It included a broad range of regression models, from logistic to linear regression to Cox proportional hazards. The study analyzed the effects of two types of errors—false positives and false negatives—in combination, as they would be expected to occur in real data. The large number of simulations utilized in the study and large datasets evaluated—tens of thousands of patients—increase reliability of our findings. Finally, the study evaluated the impact of possible NLP errors on the relationships of a broad range of variables, including demographics, comorbidities, vital signs and laboratory measurements with several different types of outcomes (laboratory tests, body measurements and receipt of treatment), increasing its generalizability.

The findings of the present study should be interpreted in the light of its limitations. Data for the analysis originated in a single integrated healthcare system in eastern Massachusetts, and therefore the findings may not be applicable to other settings. The range and types of errors simulated might not fully capture the complexity and variability of errors that occur in real-world NLP applications; errors could also vary between patient subgroups. This could limit the generalizability of the findings. Only four types of regression models were included in the analysis, and therefore the impact of possible NLP errors on other types of studies that use NLP data, such as predictive models or sentiment analysis, should be analyzed in future research. Machine learning-based NLP methods were not included in this evaluation. However, since the nature of NLP errors—false positives and false negatives—is the same irrespective of the specific NLP technique—our findings would be expected to generalize to all NLP methodologies. Finally, the studies included in this analysis focused on short to intermediate-range outcomes. Further investigations should be conducted to determine whether the impact of NLP errors could be different in analyses of epidemiology of long-term clinical outcomes.

## Conclusion

In this study that evaluated effects of NLP accuracy on a range of regression models in several real-world evidence studies, we have found that the impact of possible NLP errors on the point estimates and their statistical significance in regression models was modest. This finding supports broad use of NLP in real-world evidence investigations that utilize regression models. Further research should be conducted to assess possible impact of NLP errors on other analytical methods.

## Data Availability

This repository at https://github.com/lanzhouBWH/NLP-Downstream-Impact provides R codes (.R) and job scheduler codes (.lsf) for the Monte Carlo experiments to analyze Impact of Possible Errors in NLP-Derived Data on Downstream Epidemiologic Analysis.
